# Methods for calculating credible intervals for ratios of beta distributions with application to relative risks of death during the second plague pandemic

**DOI:** 10.1371/journal.pone.0211633

**Published:** 2019-02-22

**Authors:** Maria Bekker-Nielsen Dunbar, Thomas J. R. Finnie, Barney Sloane, Ian M. Hall

**Affiliations:** 1 Emergency Response Department, Public Health England, Porton Down, Salisbury, Wiltshire, United Kingdom; 2 Historic England, Swindon, United Kingdom; Columbia University, UNITED STATES

## Abstract

Employing historical records we are able to estimate the risk of premature death during the second plague pandemic, and identify the *Black Death* and *pestis secunda* epidemics. We show a novel method of calculating Bayesian credible intervals for a ratio of beta distributed random variables and use this to quantify uncertainty of relative risk estimates for these two epidemics which we consider in a 2 × 2 contingency table framework.

## Introduction

We present a means of calculating two forms of credible interval for ratios of binomial proportions which we use with the relative risk, a commonly used measure in medical and epidemiological research. Kawasaki and Miyaoka [1] have previously examined credible intervals for a difference between binomial proportions. We extend this work by considering ratios, such that our work can be considered an addition to the current scientific evidence on binomial proportions. Our approach is similar to that of Nurminen and Mutanen’s examination of Bayesian counterparts to frequentist tests [2]. They consider the posterior cumulative function for null hypothesis testing while we considered the ratio as being a random variable. Furthermore we extended Pham-Gia’s work on the density of such a variable by calculating the distribution on the basis of that density. Beyond this work, we prove the unimodality of this density and consider priors beyond the pairing Nurminen and Mutanen call “rectangular”, which is *α* = *β* = *θ* = *ϕ* = 1 following our notation, though we include it in our examples, which further considers non-equal priors. This means, unlike other instances found in the literature, we allow for non-symmetrical beta distributions hence it is possible to skew the priors towards probabilities considered more likely.

Our article is structured as follows: we first introduce the methods we use in our calculation, including the ratio we consider and how it arises from incidence proportions found in 2 × 2 contingency tables. Following this we calculate the credible interval. Then we introduce plague; the disease we are considering, as well as our plague data. Results are presented followed by a discussion which includes considerations surrounding our data, after which we conclude our work.

## Materials and methods

### Relative risk

The relative risk is a common epidemiological measure of association for comparison of risks of health events among different groups. In contingency tables, it is given by a ratio of incidence risk, used to compare the risk of a health event among one group, *p*, with the risk of the same health event among a second group, *q*:

$$\begin{array}{c} RR=\frac{p}{q},\;\text{for}\;p,q\in [0,1] \end{array}$$ (1)

The estimate of the relative risk for a 2 × 2 contingency table such as Table 1 is $\hat{RR}=\frac{P/M}{C/N}=\frac{PN}{MC}$, where estimates of the incidence probabilities, *p* and *q* in Eq (1), are given by the observed proportions of incidence. Conventionally, the category of interest is listed in the first row of a 2 × 2 contingency table such as Table 1.

**Table 1:** Example of 2 × 2 contingency table.

Classification	Incident		
	Did occur	Did not occur	Total at risk
Case	*P*	*M* − *P*	*M*
Control	*C*	*N* − *C*	*N*
Total	*C* + *P*	*N* − *C* + *M* − *P*	*N* + *M*

### Implementation

All calculations are performed using *Python* version 2.7.14 while graphics are made in *Python* and *R* version 3.4.1 and optimised for colour blindness according to Wong [3]. The programming language used to calculate the interval would need to be able to calculate the hypergeometric function _3_*F*_2_, as seen in the next section, which motivates our use of the programming language *Python*. See the supporting information for access to the *Python* implementation.

Determination of sensible starting values for the optimisation that calculate the credible intervals remains an open question. We have used those implemented as defaults in our implementation where possible and the values 0.4 and the relative risk estimate $\hat{RR}=0.5891$ as lower and upper starting points for the *pestis secunda* data, when default values weren’t possible.

### Credible intervals

Given a model and data, credible intervals provide us with the range of values containing the true parameter values for our parameter of interest with some pre-specified probability. We use credible intervals to quantify the uncertainty of our relative risk estimate, $\hat{RR}$, as these allow us to incorporate prior information regarding the expected distribution of deaths. We calculate these intervals using the equal-tailed quantile method and the highest posterior density method. These methods are described further below. The benefit of using a credible interval over the confidence interval is that it enables us to conclude that our parameter of interest, the relative risk, lies in the interval found with a certain probability determined beforehand, which in our case is 1 − *δ*, where we use *δ* to denotes the desired significance level to avoid confusion with the use of *α* in the beta distribution. If the credible interval contains the value 1 we may say there is no evidence of a difference between the outcomes of the groups under consideration.

**Fig 1:**
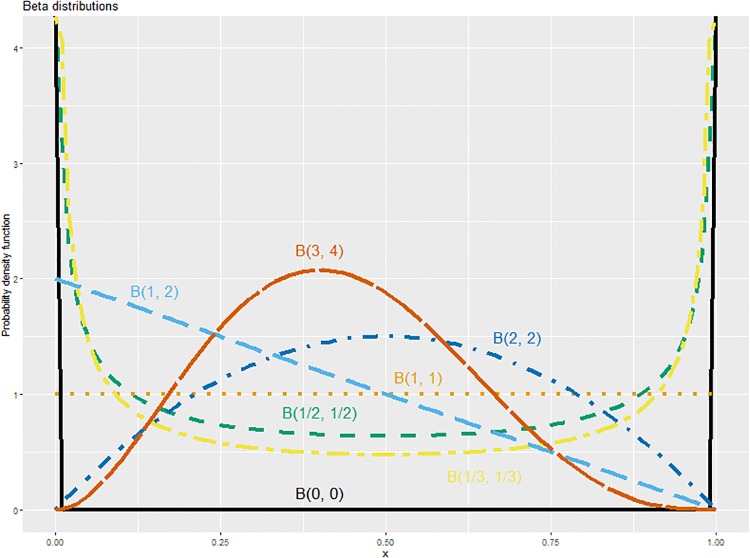
Plot of beta distributions used as priors. Probability density functions for beta distributions with various shape parameters.

We calculate credible intervals for the relative risk. We consider the incidence probabilities in our two groups to be independent binomial probabilities. This means we assume *X*_*i*_ ∼ Bin(*n*_*i*_, *p*_*i*_) for groups *i* = 1, 2. As the beta distribution is a conjugate prior for the binomial, our probabilities follow beta distributions *p*_1_ ∼ *B*(*α*, *β*) and *p*_2_ ∼ *B*(*θ*, *ϕ*). From this we obtain the posterior distributions *p*_1_∣*X*_1_ = *x*_1_ ∼ *B*(*x*_1_ + *α*, *n*_1_ − *x*_1_ + *β*) and *p*_2_ ∣ *X*_2_ = *x*_2_ ∼ *B*(*x*_1_ + *θ*, *n*_2_ − *x*_1_ + *ϕ*). For a beta distribution *B*(*α*, *β*) with shape parameters *α* and *β*, the density has more weight in the lower half when *α* < *β* while it has more weight in the upper half when *α* > *β*. When *α* = *β* the density is symmetric. The uniform prior is the special case where *α* = *β* = 1. Examples of beta distributions can be seen in Fig 1.

A posterior distribution for the probabilities (*p*_1_, *p*_2_) induces posterior distributions for the measures of association of these probabilities, including the relative risk. The distribution for the ratio is calculated as follows: Consider two independent beta distributed random variables, *X* ∼ *B*(*α*, *β*) and *Y* ∼ *B*(*θ*, *ϕ*), where *θ* and *ϕ* denote the shape parameters of the second beta distribution. Then the random variable *Z* = *Y*/*X* has density [4]

$$\begin{array}{c} f\left(z\right)=\{\begin{array}{cc} \frac{B(\alpha +\theta ,\beta )}{B(\alpha ,\beta )B(\theta ,\phi )}z^{\theta -1}{}_{2}F_{1}\left(\alpha +\theta ,1-\phi ;\alpha +\theta +\beta ;z\right), & 0<z\leq 1\\ \frac{B(\alpha +\theta ,\phi )}{B(\alpha ,\beta )B(\theta ,\phi )}z^{-(1+\alpha )}{}_{2}F_{1}(\alpha +\theta ,1-\beta ;\alpha +\theta +\phi ;\frac{1}{z}), & z>1 \end{array} \end{array}$$ (2)

from which we calculate the cumulative distribution function:

$$\begin{array}{c} F\left(z\right)=\{\begin{array}{cc} \frac{B(\alpha +\theta ,\beta )}{B(\alpha ,\beta )B(\theta ,\phi )}\frac{z^{\theta }}{\theta }{}_{3}F_{2}(1-\phi ,\alpha +\theta ,\theta ;\alpha +\theta +\beta ,\theta +1;z), & 0<z\leq 1\\ \frac{B(\theta +\alpha ,\phi )}{B(\theta ,\phi )B(\alpha ,\beta )}\frac{z^{-\alpha }}{\alpha }{}_{3}F_{2}(\theta +\alpha ,1-\beta ,\alpha ;\theta +\phi +\alpha ,\alpha +1;-\frac{1}{z}), & z>1 \end{array} \end{array}$$ (3)

**Fig 2:**
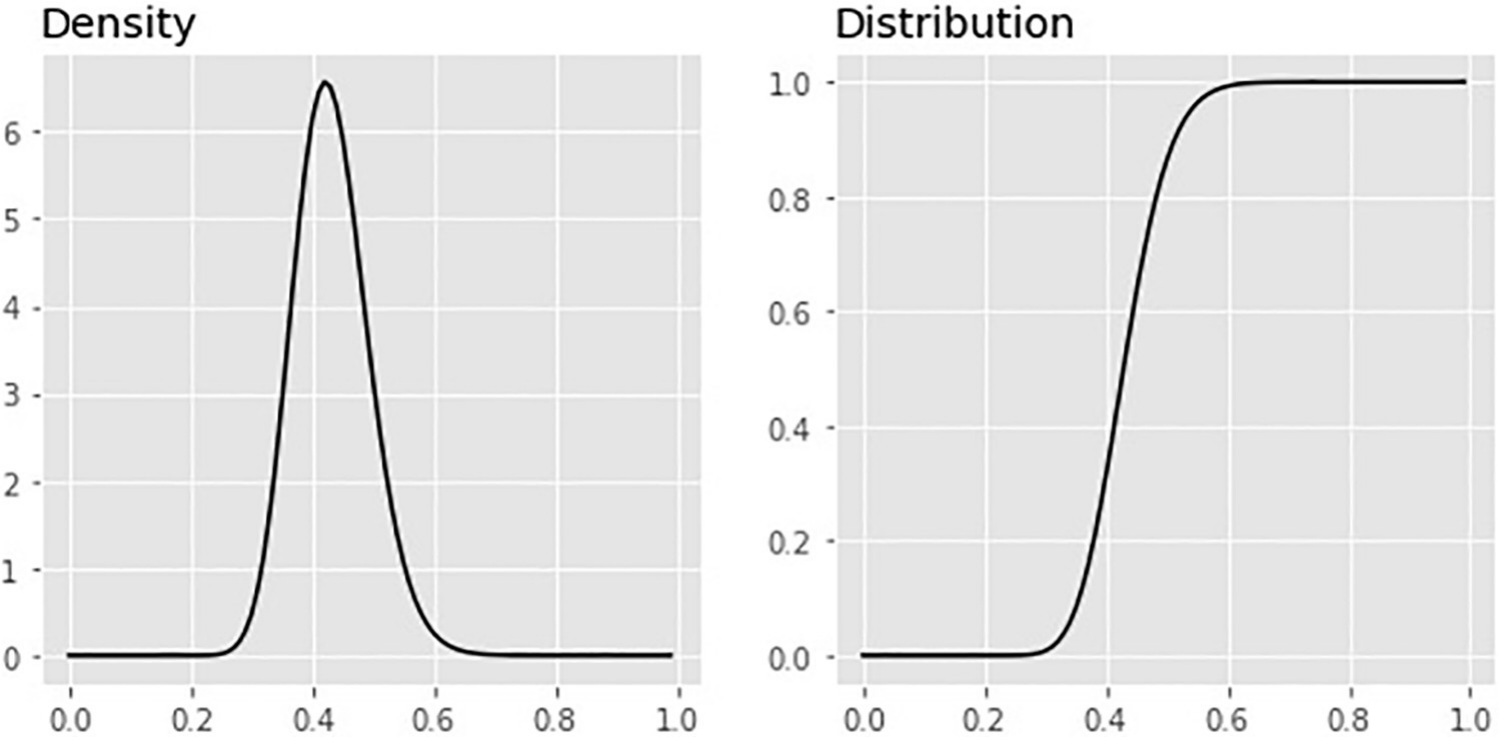
Plot of ratio density and distribution. Plot of *f*(*z*) prior density Eq (2) and *F*(*z*) posterior distribution Eq (3) for the *Black Death* plague data from Table 2 with priors *π*_1_ = *π*_2_ = *π*_3_ = *π*_4_ = 0 as an example.

Derivations can be found in the supporting information. We include our data given by *C*, *N*, *P* and *M* as in Table 1 and prior beliefs *π*_1_, *π*_2_, *π*_3_, *π*_4_ where *π*_1_ is prior belief towards *C*, *π*_2_ is prior belief towards *N* − *C*, and *π*_3_ and *π*_4_ are the corresponding beliefs for the other group. These are included by inserting *α* = *C* + *π*_1_, *β* = *N* − *C* + *π*_2_, *θ* = *P* + *π*_3_, and *ϕ* = *M* − *P* + *π*_4_. When the counts in our contingency table are large enough, the effect of the prior chosen should be small. An illustration of Eqs (2) and (3) can be found in Fig 2.

#### Beta distributions considered

We use the following pairs of priors {*B*(0, 0), *B*(0, 0)}, $\left\{B\left(\frac{1}{2},\frac{1}{2}\right),B\left(\frac{1}{2},\frac{1}{2}\right)\right\}$, $\left\{B\left(\frac{1}{3},\frac{1}{3}\right),B\left(\frac{1}{3},\frac{1}{3}\right)\right\}$, {*B*(1, 1), *B*(1, 1)}, {*B*(2, 2), *B*(2, 2)}, and {*B*(1, 2), *B*(3, 4)} in our calculations of the two types of uncertainty intervals, where {*B*(*π*_1_, *π*_2_), *B*(*π*_3_, *π*_4_)} denotes the prior values we add to our data. The probability density function of the individual beta distributions mentioned in these pairs can be found in Fig 1. We see that the individual beta distributions have very different shapes. If we have the same beliefs regarding incidence for each group, we use the same parameters in both beta distributions, i.e. *π*_1_ = *π*_3_ and *π*_2_ = *π*_4_. If the uniform prior is used, i.e. *B*(1, 1), the posterior distribution will have the same shape as the binomial likelihood function. Agresti and Min argue that using $B(\frac{1}{2},\frac{1}{2})$ performs better than using the uniform prior. They argue this based on a frequentist examination of the performance of the interval for various priors [5]. Kerman argues the default prior should be $B(\frac{1}{3},\frac{1}{3})$ since this gives rise to posterior distributions with 0.5 probability that the true parameters value is smaller or larger than the maximum likelihood estimate [6]. These arguments, in part, motivated the choices of of beta distributions used in our analysis.

#### Equal-tailed quantile interval

From the posterior distribution Eq (3) we construct our credible interval using the quantile method, solving

$$\begin{array}{c} F\left(z\right)=\{\begin{array}{c} \delta /2\\ 1-\delta /2 \end{array} \end{array}$$ (4)

to obtain the lower and upper limit of the 100(1 − *δ*)% interval, where *δ* denotes the desired significance level, whereby values contained in the interval are those between the chosen quantiles. The assumptions behind the equal-tailed quantile based interval rely on our posterior distribution being symmetric. This is trivially not an issue for the cases where the two beta distributions have the same prior values, *α* = *θ* and *β* = *ϕ*. It is up to the user of this method to examine the symmetry of the posterior distribution when using mixed beta distributions and ensure symmetry of the distribution. Where the symmetry is not assured the highest posterior density interval in the next section is suggested.

#### Highest posterior density interval

We again use the posterior density to calculate a 100(1 − *δ*)% interval, seeking intervals fulfilling

$$\begin{array}{c} P(l<z<u)=1-\delta \end{array}$$ (5)

where *l* and *u* denote the lower and upper endpoints of the interval, respectively, and *δ* is once again the desired significance level. This need not be equal-tailed as with the method in the previous section. As there are infinite intervals fulfilling Eq (5), the highest posterior density method seeks the narrowest of these intervals [7]. We have closed form expressions of *f* and *F*, given by Eqs (2) and (3) respectively, meaning we do not need to utilise kernel density estimates and can obtain the exact intervals using Chen and Shao’s Theorem 2 [8] by minimising

$$\begin{array}{c} \min_{l<u}(|f(u)-f(l)|+|F(u)-F(l)-(1-\delta )|) \end{array}$$ (6)

for *u* and *l* as *f* is continuous and unimodal. *f* is continuous due to being the product of a beta density and the reciprocal of a beta density. The beta density is itself continuous as polynomials are continous everywhere. Our density is unimodal as it has a single global maximum, we see this as our density is a function of *z*^*n*^ and _2_*F*_1_ *W*, given *z*^*n*^ is monotonically increasing or decreasing, we need _2_*F*_1_ to have a single global maximum. The conditions for this are met given appropriate parameter choices (see supporting information for details). The density is illustrated in Fig 2.

#### Comparison

The equal-tailed quantile interval works well when the posterior distribution is symmetric. When the distribution is skewed we instead use the highest posterior density interval. The highest posterior density interval can be harder to calculate as if the density considered is multimodal, the highest density region method may lead to multiple intervals, which then have to be examined to find the narrowest, though this is not the case here. The mode of the posterior distribution is contained in a highest density interval while the median is contained in the equal-tailed quantile interval. The median separates the greater and lesser halves of the density while the mode is the most frequent value in the density. Should the posterior distribution Eq (3) be symmetric, both methods are available for use, if this is not the case only the highest posterior density interval should be used.

### Data

#### Plague

Plague is a disease caused by the bacterium *Y. pestis* [9], discovered by Alexandre Yersin in 1894 [10]. It is mainly a disease of rodents and fleas [11]. Human plague epidemics are considered to occur when *Y. pestis* leaves the rodent-flea cycle [12]. There are three forms of plague when humans are infected: bubonic, where the bacillus infects the lymphatic system, septicaemic, where the bacillus infectes the bloodstream, and pneumonic when the lungs are infected by the bacillus [11]. The most common type of plague is primary bubonic, where a plague-infected flea bites a human. If the infected human develops sepsis without developing buboes, they are said to have contracted primary septicaemic plague. It is also possible to get secondary septicaemic plague following primary bubonic plague, i.e. where the sepsis occurs after buboes. Both primary bubonic and primary septicaemic plagues can lead to secondary pneumonic plague. If a human with secondary pneumonic plague comes into contact with another susceptible human, this second human may develop primary pneumonic plague, without buboes [13]. Pneumonic plague, unlike bubonic and septicaemic, can be transmitted from person to person [11]. A human can contract bubonic plague through contact with objects contaminated by sputum from a pneumonic plague sufferer [14].

There have been three documented plague pandemics throughout history. The *Black Death*, which may have come from the expression *atra mors* [15] or *pestis atra* [16] or from the acral necrosis of sufferers [13], is the colloquial name for the plague epidemic of 1348–1349. The term *Black Death* was coined 200 years after the outbreak, during the time it was called things such as *Great Dying* [12, 16], *Great Mortality* [17], and *Great Pestilence* [13, 16]. The *Black Death* was followed by another epidemic which occurred in 1361 named *pestis secunda*. We use the names *Black Death* and *pestis secunda* to distinguish between the two periods in our data, they are defined in the section on data in Materials and methods. They are considered to be the first and second wave of the second plague pandemic on the British Isles [18]. At least 18 outbreaks of plague can be identified in the period 1348 to 1485 on the British Isles [19]. It is speculated that the second plague pandemic started during the first known use of plague as a biological weapon, namely following a Tatar attack on Kaffa (today Feodosia, Ukraine) in 1346 [16, 17, 19].

#### Deaths

**Fig 3:**
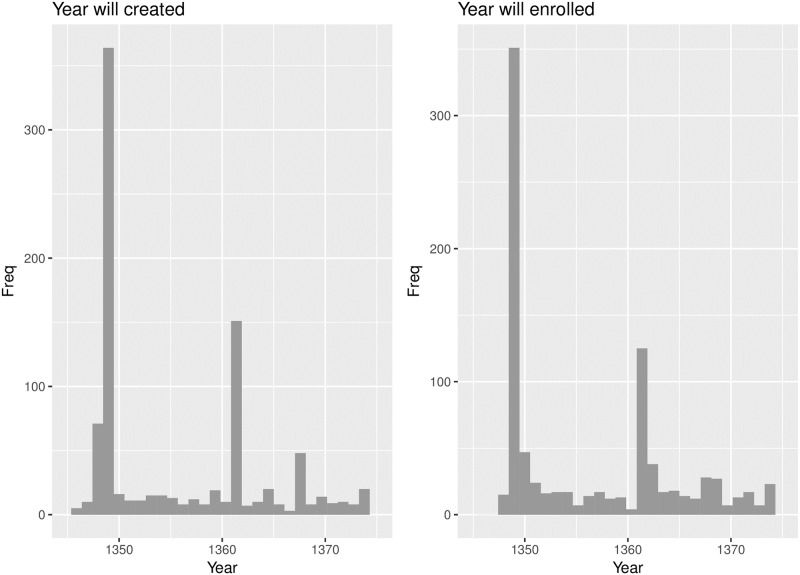
Data used. Histograms of wills created and enrolled in the data considered.

The extensive research of medieval plagues in England is due to the best records of the time being made there [18]. The second plague pandemic is well documented in comparison to earlier epidemics [17, 19]. Data for our analysis was collected in the mid to late 2000s and kindly provided by English Heritage. Data comes from testamentary records found in the Calendar of Wills in the Court of Husting, London [20, 21], including those recorded during the plague epidemics considered. A will is first proved (accepted as legally valid) in the court and then, following death, is enrolled (officially recorded) in the court. We assume that people would not create a will if they did not think they were at risk of death. From examining this data, we see a spike in number of wills created in the period from December 1348 to May 1349 both months included, henceforth referred to as the *Black Death* plague period. We observed that the number of wills enrolled follows a similar pattern to number of wills created with a slight lag. This can be seen in the histograms in Fig 3. While there will be natural lag due to wills not being created at the same time as their enrolment, we also considered the delay in wills being enrolled due to closures of the court office caused by plague. Taking this delay into account, we use a cut-off of 52 weeks, grouping people based on whether their will has taken more than a year to enrol from the date of creation.

Thus we obtain data on deaths due to plague by examining the time elapsed between a will being made and being enrolled. We obtain the counts found in Table 2 by considering numbers of dead people grouped by time elapsed prior to will enrolment and whether or not their will was made during the plague period. This time elapsed is compared for the periods December 1348 to May 1349 and January 1361 to July 1361 against the period 1347 to 1372. The estimate for the relative risk between groups 1 and 2 during the *Black Death* in Table 2 is $\hat{RR}=0.4299$. This suggests a reduced risk of dying from plague during the *Black Death* in group 1 (those surviving more than a year before dying) and thus an increased risk of $1/\hat{RR}=2.3263$ for group 2 (deaths within a year or less). There is a second spike in wills created allowing us to examine deaths from the *pestis secunda* outbreak in 1361 [18]. Using the same cut-off of 52 weeks, we again obtain data on deaths due to plague from time elapsed between a will’s creation and enrolment. This gives us the counts found in Table 3. Here this relative risk estimate is $\hat{RR}=0.5891$, giving us a slightly reduced risk for group 2 of dying from plague within a year or less compared to before with $1/\hat{RR}=1.6976$. We calculate the relative risks for the period before the *Black Death* and the period after *pestis secunda*, these are $\hat{RR}=2.5417$ and $\hat{RR}=1.0956$ respectively. These are our estimates of the non-trivial background rate of death. Their corresponding contingency tables can be found in the supporting information.

**Table 2:** Plague data for the *Black Death*.

Enrolment	In plague period		
	Yes	No	Total
More than a year (1)	56	310	366
A year or less (2)	126	228	354
Total	182	538	720

**Table 3:** Plague data for *pestis secunda*.

Enrolment	In plague period		
	Yes	No	Total
More than a year (1)	25	98	123
A year or less (2)	108	205	313
Total	133	303	436

## Results

We see that the shape of the beta distribution changes based on the parameter values. If both shape parameters are the same we obtain a symmetric distribution. *B*(0, 0) corresponds to the situation where we have no prior beliefs. *B*(1, 1) is the uniform prior as all options are equiprobable. Both *B*(0, 0) and *B*(1, 1) are improper priors. We see that when the shape parameters are between 0 and 1 we observe a U-shaped distribution. When they are larger than 1, they flip with respect to the uniform prior curve. If we consider shape parameters that are not equal, we observe asymmetric distributions, *B*(1, 2) being the most extreme case in our plot. *B*(2, 1) will act in a similar way but point in the opposite direction, producing a linear curve with slope 2. The asymmetry is not as obvious for the *B*(3, 4) case but there is a slight shift towards the left of the plot as a result of having more belief in the members of the unaffected group, causing the distribution to flatten out. Fig 1 illustrates the versatility of the beta distribution as it covers many different distribution shapes.

### Intervals

Solving each *F*(*z*) equal to our desired quantiles Eq (4), we obtain the results in Tables 4 and 5. We see that the lengths of the intervals are similar for the two epidemics though the relative risks are not that similar. Highest posterior density intervals for our relative risk can be found in Tables 6 and 7. We see once again that the lengths of the intervals are similar for the two epidemics though the relative risks are not that similar. We find similar intervals using the two methods, this is most likely due to having weak priors and sufficient data.

**Table 4:** 95% equal-tailed credible intervals for the *Black Death* plague data from Table 2 for various priors.

Prior	Lower	Upper
*B*(0, 0) and *B*(0, 0)	0.184136	0.667344
$B(\frac{1}{2},\frac{1}{2})$ and $B(\frac{1}{2},\frac{1}{2})$	0.198032	0.667712
$B(\frac{1}{3},\frac{1}{3})$ and $B(\frac{1}{3},\frac{1}{3})$	0.193495	0.667591
*B*(1, 1) and *B*(1, 1)	0.211098	0.668059
*B*(2, 2) and *B*(2, 2)	0.234964	0.668697
*B*(1, 2) and *B*(3, 4)	0.265197	0.669541

**Table 5:** 95% equal-tailed credible intervals for the *pestis secunda* plague data from Table 3 for various priors.

Prior	Lower	Upper
*B*(0, 0) and *B*(0, 0)	0.324483	0.839178
$B(\frac{1}{2},\frac{1}{2})$ and $B(\frac{1}{2},\frac{1}{2})$	0.346066	0.840491
$B(\frac{1}{3},\frac{1}{3})$ and $B(\frac{1}{3},\frac{1}{3})$	0.339108	0.840056
*B*(1, 1) and *B*(1, 1)	0.365633	0.841789
*B*(2, 2) and *B*(2, 2)	0.399598	0.844363
*B*(1, 2) and *B*(3, 4)	0.428814	0.847433

**Table 6:** 95% highest posterior density credible intervals for the *Black Death* plague data from Table 2 for various priors.

Prior	Lower	Upper
*B*(0, 0) and *B*(0, 0)	0.182746	0.848844
$B(\frac{1}{2},\frac{1}{2})$ and $B(\frac{1}{2},\frac{1}{2})$	0.187570	0.852441
$B(\frac{1}{3},\frac{1}{3})$ and $B(\frac{1}{3},\frac{1}{3})$	0.186531	0.853306
*B*(1, 1) and *B*(1, 1)	0.190038	0.852045
*B*(2, 2) and *B*(2, 2)	0.197062	0.844441
*B*(1, 2) and *B*(3, 4)	0.197427	0.866545

**Table 7:** 95% highest posterior density credible intervals for the *pestis secunda* plague data from Table 3 for various priors.

Prior	Lower	Upper
*B*(0, 0) and *B*(0, 0)	0.350345	0.866347
$B(\frac{1}{2},\frac{1}{2})$ and $B(\frac{1}{2},\frac{1}{2})$	0.381122	0.829022
$B(\frac{1}{3},\frac{1}{3})$ and $B(\frac{1}{3},\frac{1}{3})$	0.352918	0.872791
*B*(1, 1) and *B*(1, 1)	0.358073	0.8853977
*B*(2, 2) and *B*(2, 2)	0.400681	0.842793
*B*(1, 2) and *B*(3, 4)	0.373042	0.922396

## Discussion

### Death in the fourteenth century

Other causes of premature death in Great Britain at the time our data is from include famine, war with France (Hundred Years’ War), extreme weather (end of the Medieval Warm Period), and of course other diseases due to the poor hygiene and close living quarters of the time, such as dysentery and diphtheria. The medical knowledge of the time of the *Black Death* was based in humorism and miasma theory which could not cure the disease [18]. The cause of the disease was unknown at the time [19]. Some of the wills in the data mention Aldersgate, the *Black Death* cemetery founded by Walter Manny in January 1349 with a chapel consecrated in April 1349, confirming that the cause of death may have been plague. The cemetery at Aldersgate is now buried under Charterhouse Square in Smithfield. We postulate that the frequency of wills created would have increased during the plague period as people would be more likely to write a will if they feared they might die, which is why we can deduce cause of death to likely be the plague. Since the *Black Death* was the major killer of the time, we conclude the deaths contained in our data recorded in the period considered are most likely due to the second plague pandemic.

### Reoccurrences of plague

We hypothesise that the delay prior to the second spike seen in the data is due to the cyclical nature of the second plague pandemic. With more data we could examine the gap between outbreaks more thoroughly. It should be noted that Roll 88 of the court our data is sourced from is missing which covers part of 1360 to February 1361. This means that our data for *pestis secunda* may be sparser that it need be. *pestis secunda* is also known as *pestis puerorum*; “children’s plague” [18]. This is due to it mainly affecting children who had not been alive during the *Black Death*. So it could have been the case that the children who were not exposed to the plague during the *Black Death* did not have the same immunity as the people who survived the *Black Death*. It is worth noting that the skeletal make-up of the cemetery at East Smithfield, excavated in 1986 and 1987, argued to be that from 1361, see [22], suggested no real variation in juvenile death rates compared with the larger 1349 cemetery on the same site. If this is accepted, the reports that *pestis secunda* affected children excessively might need to be viewed with some scepticism. The *Black Death* was a combination of bubonic, pneumonic and septicaemic plague, with bubonic being the most common, while the *pestis secunda* epidemic is believed to primarily have been caused by bubonic plague [18] which might also have influenced the spread of disease. Though this could also have been due to seasonal variation, we do not have climate data available to examine whether the climate was significiantly different during the two epidemics.

Alternatively, humans are considered tertiary hosts of the plague bacteria *Y. pestis*, following the secondary hosts: rats, at the time most likely *Rattus rattus*, and primary hosts: fleas, meaning humans are secondary epizootic victims of silvatic plague. When silvatic plague occurs in a rodent population, a reservoir of plague arises which may explain the cyclic behaviour, further more *Y. pestis* can survive in burrows after epizootic extermination of rodents which leads to a revival of plague when the next rodent population occupied the burrow [18]. Hence there may have been a depletion of the rat population during the period between the two outbreaks considered, this could be following their plague epizootic which is assumed to have occurred prior to the plague epidemics as the fleas would seek other hosts; humans. It has also been suggested that instead of transmission from *Xenopsylla cheopis* fleas from rats to humans, transmission might have occured via humans through the ectoparasites *Pulex irritans* (flea) or *Pediculus humanus humanus* (louse) [16, 23, 24]. The rat flea could also have been *Nosopsyllus fasciatus* instead of *X. cheopis* due to the climate of England [25]. Medics of the time are not noted to have observed the connection between dead rodents preceeding an outbreak.

Following *Black Death* and *pestis secunda*, a plague cycle has been observed by historians, hence these epidemics are considered part of a plague pandemic. The cyclic outbreaks of plague occurred every 5-12 years during the second pandemic [18]. Indeed, we can see a small spike in our data in the right of the plots in Fig 3 for *pestis tertia*, the third epidemic (ca. 1369 there are eight records of wills being made in that year in our data). If we collected data covering later years we could also examine *pestis quarta*, the fourth epidemic (ca. 1375), and *pestis quinta*, the fifth epidemic (ca. 1379-1383). Our current data is too sparse to examine these epidemics, however with further evidence we could potentially confirm the cyclic nature of the second plague pandemic. The names of epidemics used here to distinguish the various outbreaks are those mentioned used by English annalists from Creighton [26].

## Conclusion

We considered the ratio of two beta distributions and calculated a closed form expression of the posterior distribution of such a ratio. To consider the uncertainty of relative risks, we considered two types of credible interval, the quantile interval and the highest posterior density. We saw that in the situation considered they gave similar results and did not contain 1 indicating that risk of death due to plague was significantly different to non-plague death in the periods we considered. We saw a reduced risk of dying from plague during the *pestis secunda* outbreak comapred to the risk of dying from plague during the *Black Death* outbreak.

Applications of our prior density Eq (2) and posterior distribution Eq (3) are not constrained to relative risks, they can be used on any ratio of probabilities if we can justify assuming that those probabilities arise from binomial distributions. In terms of counts, we can use it in any situation where we have point estimates based on proportions, meaning it is not limited to the use of ratios from 2 × 2 contingency tables. Our methods can be considered general as we have considered general priors (*α*, *β*, *θ*, *ϕ*) which do not necessarily have to match to obtain symmetry, due to our highest posterior density calculations, unlike other methods seen in the literature. Our method is avaliable for use at https://www.github.com/PublicHealthEngland/bayesint. For more information, see the supporting information.

## Supporting information

S1 File Additional calculations. (PDF)

S2 File Code used to create image in S1 File. (R)
