# Supplementary material for: Methods for calculating credible intervals for ratios of beta distributions with application to relative risks of death during the second plague pandemic
Source: PLoS One. 2019 Feb 22;14(2):e0211633. doi: 10.1371/journal.pone.0211633 (PMC6386284; doi:10.1371/journal.pone.0211633)
Supplement: S1 File — (PDF) [file pone.0211633.s001.pdf]

Supporting information for Methods for calculating credible intervals for ratios of beta distributions with application to relative risks of death during the second plague pandemic

Maria Bekker-Nielsen Dunbar[1*], Thomas J. R. Finnie[1], Barney Sloane[2], Ian M. Hall[1¤]

1 Emergency Response Department, Public Health England, Porton Down, Salisbury, Wiltshire, United Kingdom
2 Historic England, Swindon, United Kingdom

¤Current Address: University of Manchester, Manchester, United Kingdom
* maria.dunbar@phe.gov.uk (MBND)

1 Bayesint Python module

The *Python* module used to calculate the numbers in this manuscript is hosted at and can be installed from the bayesint repository at Public Health England's Github account `github.com/PublicHealthEngland/bayesint`.

2 Cumulative distribution function

We calculate $F(z)$ based on the density Eq (2) from the main text. We obtain the following cumulative distribution function in the case where $z > 1$.

$$\begin{aligned} F(z) &= \int_{-\infty}^{z} f(x)\,dx = \int_{0}^{z} f(x)\,dx \\ &= \frac{B(\theta+\alpha,\phi)}{B(\theta,\phi)B(\alpha,\beta)} \int_0^z x^{-(1+\alpha)}\,{}_2F_1\left(\theta+\alpha, 1-\beta; \theta+\alpha+\phi; \frac{1}{x}\right) dx \\ &= \frac{B(\theta+\alpha,\phi)}{B(\theta,\phi)B(\alpha,\beta)} \sum_{i=0}^{\infty} \frac{(\theta+\alpha)_i(1-\beta)_i}{(\theta+\phi+\alpha)_i\Gamma(i+1)} \int_0^z x^{-(1+\alpha+i)}\,dx \\ &= \frac{B(\theta+\alpha,\phi)}{B(\theta,\phi)B(\alpha,\beta)} \sum_{i=0}^{\infty} \frac{(\theta+\alpha)_i(1-\beta)_i}{(\theta+\phi+\alpha)_i\Gamma(i+1)} \left(-\frac{z^{-(\alpha+i)}}{\alpha+i}\right) \qquad \text{Eq (1)} \\ &= \frac{B(\theta+\alpha,\phi)}{B(\theta,\phi)B(\alpha,\beta)} \sum_{i=0}^{\infty} \frac{(\theta+\alpha)_i(1-\beta)_i}{(\theta+\phi+\alpha)_i\Gamma(i+1)} \frac{(\alpha)_i}{(\alpha+1)_i\alpha} \left(-z^{-(\alpha+i)}\right) \\ &= \frac{B(\theta+\alpha,\phi)}{B(\theta,\phi)B(\alpha,\beta)} \frac{z^{-\alpha}}{\alpha}\,{}_3F_2\left(\theta+\alpha, 1-\beta, \alpha; \theta+\phi+\alpha, \alpha+1; -\frac{1}{z}\right) \end{aligned}$$

where $(x)_i$ denotes the rising factorial, given by

$$(x)_i = \frac{\Gamma(x+i)}{\Gamma(x)} \qquad \text{Eq (2)}$$

While this is traditionally denoted $(x)^{(i)}$ and the Pochhammer symbol is used for the falling factorial, we are using the notation common to hypergeometric functions.

Likewise for $0 < z \leq 1$, we obtain

$$\begin{aligned} F(z) &= \int_0^z f(x)\,dx \\ &= \frac{B(\alpha+\theta,\beta)}{B(\theta,\phi)B(\alpha,\beta)} \int_0^z x^{\theta-1}\,{}_2F_1\left(\theta+\alpha, 1-\phi; \theta+\alpha+\beta; x\right) dx \qquad \text{Eq (3)} \\ &= \frac{B(\alpha+\theta,\beta)}{B(\alpha,\beta)B(\theta,\phi)} \frac{z^\theta}{\theta}\,{}_3F_2\left(1-\phi, \alpha+\theta, \theta; \alpha+\theta+\beta, \theta+1; z\right) \end{aligned}$$

We add priors to our observed values by inserting $\alpha = C + \pi_1$, $\beta = N - C + \pi_2$, $\theta = P + \pi_3$, and $\phi = M - P + \pi_4$.

3 Mean and variance of distribution

We calculate the mean, $E(Z)$, and variance, $Var(Z)$, of our distribution for Z by

$$E(Z) = \int_{-\infty}^{\infty} z f(z)\,dz \qquad \text{Eq (4)}$$

and

$$Var(Z) = \int_{-\infty}^{\infty} z^2 f(z)\,dz - \left(\int_{-\infty}^{\infty} z f(z)\,dz\right)^2 \qquad \text{Eq (5)}$$

Due to the cases in Eq (2) from the main text and $z > 0$, we have

$$\begin{aligned}
E(Z) &= \int_{-\infty}^{\infty} zf(z)\,dz = \int_0^1 zf(z)dz + \int_1^{\infty} zf(z)\,dz \\
&= \frac{B(\alpha+\theta,\beta)}{B(\alpha,\beta)B(\theta,\phi)}\int_0^1 z^{\theta}{}_2F_1(\alpha+\theta,1-\phi;\alpha+\theta+\beta;z)\,dz \\
&\quad + \frac{B(\alpha+\theta,\phi)}{B(\alpha,\beta)B(\theta,\phi)}\int_1^{\infty} z^{-\alpha}{}_2F_1\left(\alpha+\theta,1-\beta;\alpha+\theta+\phi;\frac{1}{z}\right)dz \\
&= \frac{B(\alpha+\theta,\beta)}{B(\alpha,\beta)B(\theta,\phi)}\sum_{i=0}^{\infty}\frac{(\alpha+\theta)_i(1-\phi)_i}{(\alpha+\theta+\beta)_i\Gamma(i+1)}\int_0^1 z^{\theta+i}\,dz \\
&\quad + \frac{B(\alpha+\theta,\phi)}{B(\alpha,\beta)B(\theta,\phi)}\sum_{i=0}^{\infty}\frac{(\alpha+\theta)_i(1-\beta)_i}{(\alpha+\theta+\phi)_i\Gamma(i+1)}\lim_{n\to\infty}\int_1^{n} z^{i-\alpha}\,dz \\
&= \frac{B(\alpha+\theta,\beta)}{B(\alpha,\beta)B(\theta,\phi)}\sum_{i=0}^{\infty}\frac{(\alpha+\theta)_i(1-\phi)_i}{(\alpha+\theta+\beta)_i\Gamma(i+1)}\left[\frac{z^{\theta+i+1}}{\theta+i+i}\right]_{z=0}^{z=1} \\
&\quad + \frac{B(\alpha+\theta,\phi)}{B(\alpha,\beta)B(\theta,\phi)}\sum_{i=0}^{\infty}\frac{(\alpha+\theta)_i(1-\beta)_i}{(\alpha+\theta+\phi)_i\Gamma(i+1)}\lim_{n\to\infty}\left[\frac{z^{i-\alpha+1}}{i-\alpha+1}\right]_{z=1}^{z=n} \qquad \text{Eq (6)} \\
&= \frac{B(\alpha+\theta,\beta)}{B(\alpha,\beta)B(\theta,\phi)}\sum_{i=0}^{\infty}\frac{(\alpha+\theta)_i(1-\phi)_i}{(\alpha+\theta+\beta)_i\Gamma(i+1)}\frac{1^{\theta+i+1}}{\theta+i+1} \\
&\quad + \frac{B(\alpha+\theta,\phi)}{B(\alpha,\beta)B(\theta,\phi)}\sum_{i=0}^{\infty}\frac{(\alpha+\theta)_i(1-\beta)_i}{(\alpha+\theta+\phi)_i\Gamma(i+1)}\lim_{n\to\infty}\frac{n^{i-\alpha+1}-1^{i-\alpha+1}}{i-\alpha+1} \\
&= \frac{B(\alpha+\theta,\beta)}{B(\alpha,\beta)B(\theta,\phi)}\sum_{i=0}^{\infty}\frac{(\alpha+\theta)_i(1-\phi)_i}{(\alpha+\theta+\beta)_i\Gamma(i+1)}1^i\frac{(\theta+1)_i}{(\theta+2)_i(\theta+1)} \\
&\quad + \frac{B(\alpha+\theta,\phi)}{B(\alpha,\beta)B(\theta,\phi)}\sum_{i=0}^{\infty}\frac{(\alpha+\theta)_i(1-\beta)_i}{(\alpha+\theta+\phi)_i\Gamma(i+1)}(0^i-1^i)\frac{(1-\alpha)_i}{(2-\alpha)_i(1-\alpha)} \\
&= \frac{B(\alpha+\theta,\beta)}{B(\alpha,\beta)B(\theta,\phi)}{}_3F_2(\alpha+\theta;1-\phi;\theta+1;\alpha+\theta+\beta,\theta+2;1)\frac{1}{\theta+1} \\
&\quad + \frac{B(\alpha+\theta,\phi)}{B(\alpha,\beta)B(\theta,\phi)}\left(\frac{1}{1-\alpha} - {}_3F_2(\alpha+\theta,1-\beta;1-\alpha;\alpha+\theta+\phi;2-\alpha;1)\frac{1}{1-\alpha}\right)
\end{aligned}$$

since ${}_3F_2$ takes the value 1 when the third argument is 0. We similarly calculate the variance and obtain

$$\begin{aligned}
Var(Z) &= \int_{\infty}^{\infty} z^2 f(z)\,dz - \left(\int_{\infty}^{\infty} zf(z)\,dz\right)^2 \\
&= \frac{B(\alpha+\theta,\beta)}{B(\alpha,\beta)B(\theta,\phi)}{}_3F_2(\alpha+\theta,1-\phi,\theta+2;\alpha+\theta+\beta,\theta+3;1)\frac{1}{\theta+2} \qquad \text{Eq (7)} \\
&\quad + \frac{B(\alpha+\theta,\phi)}{B(\alpha,\beta)B(\theta,\phi)}\left(\frac{1}{2-\alpha} - {}_3F_2(\alpha+\theta,1-\beta,2-\alpha;\alpha+\theta+\beta,3-\alpha;1)\frac{1}{2-\alpha}\right) \\
&\quad - \left(\frac{B(\alpha+\theta,\beta)}{B(\alpha,\beta)B(\theta,\phi)}{}_3F_2(\alpha+\theta;1-\phi;\theta+1;\alpha+\theta+\beta,\theta+2;1)\frac{1}{\theta+1}\right. \\
&\quad \left. + \frac{B(\alpha+\theta,\phi)}{B(\alpha,\beta)B(\theta,\phi)}\left(\frac{1}{1-\alpha} - {}_3F_2(\alpha+\theta,1-\beta;1-\alpha;\alpha+\theta+\phi;2-\alpha;1)\frac{1}{1-\alpha}\right)\right)^2
\end{aligned}$$

4 Background rates of death

We consider the background rates of time to will enrolment by considering the wills in our data from before December 1348 and after July 1361 against those counts from the period 1347 to 1372. These can be found in Tables 1 and 2. The estimate of the relative risk for before is $\hat{RR} = 2.5417$ and the estimate of the relative risk for after is $\hat{RR} = 1.0956$.

Table 1. Before *Black Death.*

Enrolment	Before plague		
	Yes	No	Total
More than a year (1)	38	29	67
A year or less (2)	189	658	847
Total	227	687	914

Table 2. After *pestis secunda.*

Enrolment	After plague		
	Yes	No	Total
More than a year (1)	52	143	195
A year or less (2)	175	544	719
Total	227	687	914

5 Proof of unimodality of density

We consider the case where $RR = PN/MC < 1$ without loss of generality. This means that $C/N > P/M$. Moreover $\alpha = C + \pi_1$, $\theta = P + \pi_3$, $\beta = N - C + \pi_2$ and $\phi = M - P + \pi_4$.

The function

$$f(z; \alpha, \beta, \theta, \phi) = \frac{B(\alpha+\theta; \phi)}{B(\alpha; \beta)B(\theta; \phi)} z^{-(1+\alpha)}\, {}_2F_1\left(1-\beta, \alpha+\theta; \alpha+\theta+\phi; \frac{1}{z}\right) \qquad \text{Eq (8)}$$

is valid when $z \geq 1$ has the following limiting values

$$\begin{aligned} f_1^+ &= \lim_{z\to 1} f(z; \alpha, \beta, \theta, \phi) \\ &= \frac{B(\alpha+\theta; \phi)}{B(\alpha; \beta)B(\theta; \phi)} \frac{\Gamma(\alpha+\theta+\phi)\Gamma(\phi+\beta-1)}{\Gamma(\phi)\Gamma(\alpha+\theta+\phi+\beta-1)} = \frac{B(\alpha+\theta, \phi+\beta-1)}{B(\alpha; \beta)B(\theta; \phi)} \end{aligned} \qquad \text{Eq (9)}$$

([1] eqn 15.1.20) and $f_\infty = \lim_{z\to\infty} f(z; \alpha, \beta, \theta, \phi) = 0$. Our data are counts and we can demand that $\beta + \phi - 1 = N - C + \pi_2 + M - P + \pi_4 - 1 > 0$ (the most extreme case is that $C = N$ but in this situation $P \neq M$ due to $C/N > P/M$ (there must be some response in positive group), so if $P = M - 1$ this demands $\pi_2 + \pi_4 > 0$ (there must be some prior) or if $\pi_2 = \pi_4 = 0$ is necessary then $P + 1 < M$; in all other parametrer sets the condition holds) so that $f_1^+ > f_\infty$. For values of $z > 1$ noting $\alpha > 0$ we have $z^{-n-\alpha-1}$ (where n is the index from the series definition of ${}_2F_1$) decreasing and so for increasing values of z the function is montonically decreasing. If $RR > 1$ then the problem can be recast defining $C' = N - C$ as the observed count for the outcome group of interest.

In the other case, $z \le 1$, the function

$$f(z;\alpha,\beta,\theta,\phi) = \frac{B(\alpha+\theta;\beta)}{B(\alpha;\beta)B(\theta;\phi)} z^{\theta-1} \, {}_2F_1\left(1-\phi, \alpha+\theta; \alpha+\theta+\beta; z\right)$$

is valid when $z \le 1$. In order to prove unimodality, we show that the first derivative changes sign in range $[0,1]$ and that the second derivative is negative for all $z \in [0,1]$. The derivative of f is given by

$$\begin{aligned} f'(z;\alpha,\beta,\theta,\phi) &= \frac{df(z;\alpha,\beta,\theta,\phi)}{dz} = \frac{B(\alpha+\theta;\beta)}{B(\alpha;\beta)B(\theta;\phi)} \left[(\theta-1)z^{\theta-2}\right. \\ & {}_2F_1\left(1-\phi,\alpha+\theta;\alpha+\theta+\beta;z\right) + \frac{(1-\phi)(\alpha+\theta)}{\alpha+\theta+\beta} z^{\theta-1} \\ & \left. {}_2F_1\left(2-\phi,\alpha+\theta+1;\alpha+\theta+\beta+1;z\right)\right] \end{aligned} \qquad \text{Eq (10)}$$

which attains its maximum when $z = z_m$ which is given by

$$\frac{(\phi-1)(\alpha+\theta)}{(\theta-1)(\alpha+\theta+\beta)} z_m = \frac{{}_2F_1\left(1-\phi,\alpha+\theta;\alpha+\theta+\beta;z_m\right)}{{}_2F_1\left(2-\phi,\alpha+\theta+1;\alpha+\theta+\beta+1;z_m\right)}. \qquad \text{Eq (11)}$$

Moreover if the right hand side of Eq (11) is monotonically decreasing then there will only be one root.

$$\frac{{}_2F_1\left(1-\phi,\alpha+\theta;\alpha+\theta+\beta;z_m\right)}{{}_2F_1\left(2-\phi,\alpha+\theta+1;\alpha+\theta+\beta+1;z_m\right)} = \frac{{}_2F_1\left(\beta,\alpha+\theta+\beta+\phi-1;\alpha+\theta+\beta;z_m\right)}{{}_2F_1\left(\beta,\alpha+\theta+\beta+\phi-1;\alpha+\theta+\beta+1;z_m\right)}(1-z_m \qquad \text{Eq (12)}$$

([1] 15.3.3) which means we can write Eq (11) as

$$A\frac{z_m}{1-z_m} = \frac{{}_2F_1(a,b;c;z_m)}{{}_2F_1(a,b;c+1;z_m)} \qquad \text{Eq (13)}$$

where $a = \beta$, $b = \alpha+\theta+\beta+\phi-1$, and $c = \alpha+\theta+\beta$ and the left hand side monotonically increases from 0 to $+\infty$ on the range $z \in [0,1]$. The parameters of the ${}_2F_1$ hypergeometric function on the right hand side are all positive thus the ratio must decrease monotonically and so there can be only one maximum on the range. Hence, our density is unimodal and we can use the approach from Chen and Shao.

6 Confidence intervals

The Wald confidence interval [2] for the logarithm of the relative risk $\log(RR)$ by Katz [3]. The confidence interval for the relative risk is calculated by

$$\log(\hat{RR}) \pm z_{\delta/2}\hat{se}(\log(\hat{RR})) \qquad \text{Eq (14)}$$

where

$$\hat{se}(\log(\hat{RR})) = \sqrt{\frac{1}{P} + \frac{1}{C} - \frac{1}{M} - \frac{1}{N}} \qquad \text{Eq (15)}$$

and $z_{\delta/2}$ is the $100(1-\delta/2)$ quantile of the standard normal distribution, where δ denotes the desired significance level. Such an interval, based on the asymptotic behaviour of $\log(RR)$, allows us to examine the performance of the confidence interval against our credible interval, which does not have random endpoints. In some instances,

we expect there to be little difference between the credible intervals and the log Katz interval. Two other commonly used confidence intervals for ratios of proportions are the inverse hyperbolic sine interval and the score test confidence interval. The inverse hyperbolic sine interval was introduced by Newcombe [4] and calculated for the relative risk by Price and Bonett [5]. Here we calculate

$$\log(\hat{RR}) \pm 2\sinh^{-1}\left(\frac{z_{\delta/2}}{2}\hat{se}(\log(\hat{RR}))\right) \qquad \text{Eq (16)}$$

using the same estimate for $\hat{se}(\log(\hat{RR}))$ as in the log Katz interval. The score test confidence interval is attributed to Koopman [6]. We consider X and Y to be independent binomially distributed random variables with $X \sim Bin(\pi_1, n_1)$ and $Y \sim Bin(\pi_2, n_2)$. The score is given by

$$U_{RR}(x,y) = \frac{(x - n_1 p_1)^2}{n_1 p_1 (1-p_1)}\left(1 + \frac{n_1(RR - p_1)}{n_2(1-p_1)}\right), \qquad \text{Eq (17)}$$

where

$$p_1 = \frac{RR(n_1+y) + x + n_2 - ((RR(n_1+y)+x+n_2)^2 - 4RR(n_1+n_2)(x+y))^{1/2}}{2(n_1+n_2)} \qquad \text{Eq (18)}$$

and

$$p_2 = \frac{p1}{RR} \qquad \text{Eq (19)}$$

and we consider the region

$$\{RR \mid U_{RR}(x,y) \leq \chi^2_{1,1-\delta}\} \qquad \text{Eq (20)}$$

It is consistent with a Pearson χ^2 test for a 2×2 table [7].

Our reason for chosing to use credible intervals in our analysis is that they allow us to use the conditionality principle to take the wills data into account in our analysis. We only have the one data set and will not be able to obtain data from the entire sample space, i.e. on all possible repetitions of the natural experiment it arose from (plague outbreaks in London, England in the 1300s), which is what confidence intervals concern. An example of what some confidence intervals look like compared to our credible intervals for the Black Death data can be seen in Fig 1.

Fig 1. Plot of confidence and credible intervals. Confidence and credible intervals for the Black Death data, including the highest posterior density (HPD) interval.

References

1. Abramowitz M, Stegun IA. Handbook of Mathematical Functions with Formulas, Graphs, and Mathematical Tables. United States Department of Commerce, National Bureau of Standards 1972.

2. Wald, A. Tests of Statistical Hypotheses Concerning Several Parameters when the Number of Observations is Large. Trans Am Math Soc. 1943;54:426–482.

3. Katz D, Baptista J, Azen S, Pike M. Obtaining Confidence Intervals for the Risk Ratio in Cohort Studies. Biometrics. 1978;34(3):469–474.

4. Newcombe R G. Logit Confidence Intervals and the Inverse Sinh Transformation. Am Stat. 2001;55(3):200–202.

5. Price, R M, Bonett D G. Confidence intervals for a ratio of two independent binomial proportions. Statist Med. 2008;27:5497–5508.

6. Koopman P. Confidence Intervals for the Ratio of Two Binomial Proportions. Biometrics. 1984;40(2):513–517.

7. Gart J, Nam J. Approximate Interval Estimation of the Ratio of Binomial Parameters: A Review and Corrections for Skewness. Biometrics. 1988;44(2):323–338.
